# Dietary Flaxseed Oil and Its Blended Oil Alleviate High-Fat Diet-Induced Obesity in Mice by Improving Lipid Metabolism and Regulating Gut Microbiota

**DOI:** 10.3390/foods14111877

**Published:** 2025-05-26

**Authors:** Haizhen Li, Mingyue Shen, Xianxiang Chen, Yi Wu, Fengjiao Zeng, Jianhua Xie

**Affiliations:** State Key Laboratory of Food Science and Resources, Nanchang University, No. 235 Nanjing East Road, Nanchang 330047, China; lihaizhen0302@163.com (H.L.); xianxiangchen@email.ncu.edu.cn (X.C.); ah_wuyi@163.com (Y.W.); zfj2405yeah@163.com (F.Z.)

**Keywords:** flaxseed oil, blended oil, diet-induced obesity, lipid metabolism, liver damage, gut microbiota

## Abstract

Obesity represents a chronic metabolic disorder feature by dysregulated glucose-lipid homeostasis. We investigated the effects of flaxseed oil (FO), rich in α-linolenic acid, and its blended oil (BO) on high-fat diet-induced obese mice. In the BO, the mass ratio of flaxseed oil, sunflower oil (as a source of linoleic acid), and olive oil (as a source of oleic acid) was precisely set at 11.90:51.64:36.46 (*w*/*w*/*w*) After 13 weeks of supplementation, both FO and BO significantly suppressed weight gain (multiple comparisons of weight gain on week 13: 8.57 ± 1.25 g in the ND group; 25.08 ± 2.96 g in the HFD group; 19.35 ± 1.47 g/19.71 ± 2.96 g in the HFD+FO/HFD+BO group), fat accumulation, and restored dyslipidemia (notably, FO administration resulted in a significant reduction in LDL-C and LEP levels (*p* < 0.01)), elevated blood glucose (FO demonstrated a more pronounced effect compared to BO), and liver tissue damage (specifically, FO exhibited a more pronounced effect in decreasing the levels of oxidative stress markers, including alanine aminotransferase (ALT) and malondialdehyde (MDA), and BO demonstrated greater efficacy in ameliorating the histopathological conditions of liver tissue) in HFD-fed mice. The 16S rRNA gene sequencing of mice fecal samples showed that FO and BO reduced the Firmicutes/Bacteroidetes (F/B) ratio (supplementation with FO decreased the F/B ratio from 68.95 to 15.24 (*p* < 0.01), while BO supplementation reduced it from 68.95 to 19.47), decreased the abundance of Proteobacteria (supplementation with FO decreased the abundance of Proteobacteria from 0.21 to 0.15, whereas supplementation with BO reduced it to 0.17). In addition, FO increased the abundance of *Clostridium*, and BO increased the abundance of *Lactobacillus* (rose from 5.42 to 10.3), reversing the imbalance of gut microbiota in obese mice. These findings suggest that FO and BO may be promising dietary strategies for treating obesity and improving its associated metabolic disorders.

## 1. Introduction

Flaxseed oil (FO) represents a significant dietary source of essential fatty acids, particularly alpha-linolenic acid (ALA), an omega-3 (ω-3) polyunsaturated fatty acid (PUFA) that constitutes 43.8–70.0% of its total lipid profile [1]. Scientific evidence demonstrates that the bioactive compounds in FO, notably ALA and other minor constituents, exhibit diverse physiological benefits encompassing antioxidant, anti-inflammatory, cardioprotective, and lipid-modulating properties [2]. Despite these advantages, the inherent fatty acid distribution in unblended FO fails to achieve the optimal ω-6/ω-3 balance recommended by WHO guidelines [3]. The metabolic interplay between these essential fatty acids necessitates careful regulation, as their equilibrium significantly influences various biological processes [4]. Furthermore, the sensory attributes of FO are compromised by bitter peptides that generate undesirable organoleptic characteristics, potentially limiting its widespread consumption [5]. Strategic formula design using complementary vegetable oils to create blended oil (BO) is a feasible solution for simultaneously optimizing fatty acid composition and improving palatability. Specifically, combining sunflower oil (rich in linoleic acid) with olive oil (high in oleic acid) [6] offers an economically feasible approach to attain both WHO-compliant PUFA ratios and enhanced sensory qualities while maintaining cost-effectiveness [7].

Obesity represents a metabolic disorder marked by the accretion of body fat and dyslipidemia in blood glucose. This pathological condition is strongly correlated with the development of numerous systemic complications and significantly elevates the risk for various chronic disorders, particularly NAFLD and type 2 diabetes mellitus [8,9]. Global obesity rates have experienced an approximate threefold increase since the mid-1970s, with an estimated 2.5 billion (43%) adults globally overweight in 2022, of whom 890 million (16%) are obese [10]. Obesity has arisen as a major worldwide health crisis and a serious economic burden due to the increasing number of overweight and obese people [11].

Studies have found that the gut microbiome is fundamentally intertwined with obesity [12]. Both animal and epidemiological studies have confirmed that substantial dysregulations in the gut microbial community and its associated metabolic pathways were significantly elevated, acting as major hallmarks of obesity and metabolic dysfunction [13,14]. Obesity can lead to significant changes in the intestinal microbiota, the most prominent feature of which is the decrease in the α-diversity index (Shannon index and Chao1 index), a substantial elevation in the Firmicutes over Bacteroidetes ratio, and a marked augmentation in the count of Proteobacteria contrasted with the normal metabolic control group [15,16]. Wang et al. [17] demonstrated that this metabolic state disrupts gut microbiota metabolic cross-talk, particularly through the depletion of short-chain fatty acid (SCFA)-producing symbionts and the accumulation of endotoxin-generating pathobionts. Notably, microbially derived SCFAs exert pleiotropic protective effects via G protein-coupled receptor (GPCR) signaling pathways, ameliorating adipose tissue inflammation and enhancing insulin sensitivity through the epigenetic modulation of PPARγ (Peroxisome Proliferator-Activated Receptor Gamma) coactivator-1α. Dietary interventions can modulate gut microbiota, re-establishing microecological balance and exerting synergistic therapeutic effects in improving obesity and metabolic disorders.

It is well known that a high-fat diet (HFD) plays a key role in obesity, especially the excessive intake of saturated fatty acids [18]. However, obese patients often exhibit unhealthy eating habits, such as a preference for fried foods and desserts, and these habits are notoriously difficult to modify. [19]. Changing the types of dietary fats (rather than reducing the total fat intake) might offer a feasible strategy that is in line with existing dietary preferences and can improve metabolic health. The strategic replacement of saturated fatty acids with polyunsaturated fatty acids (PUFAs) may be a perfect alternative [20]. The excessive intake of lard and palm oil has been reported to cause obesity, whereas HFD prepared with flaxseed oil does not result in obesity [21]. It has been shown that ω-3 PUFAs can ameliorate obesity by regulating lipid metabolism, modulating adipokine secretion, and reducing visceral fat area [22]. FO is a high-quality provider of ω-3 PUFAs, with a particularly high content of α-linolenic acid [23]. Baranowski et al. [24] showed that dietary FO supplementation demonstrated substantial effects in obese Zucker rats, including a reduction in adipocyte hypertrophy, the downregulation of inflammatory mediators (MCP-1 and TNF-α), and decreased T-cell infiltration in adipose tissue. Yang et al. [25] found that FO refined gut microbiota dysbiosis by increasing the diversity and abundance of the *Clostridium* and Ruminococcaceae communities, thus alleviating obesity. However, whether BO also has the ability to ameliorate obesity is unclear.

Therefore, the present study comprehensively evaluated the effects of the long-term intake of FO and BO on HFD-induced obesity in mice. We systematically investigated the physiological mechanisms of FO and BO in obesity intervention through multidimensional assessments, including the following: (1) Systemic physiological parameters (body weight dynamics, lipid profiles, glucose homeostasis, serum biochemistry) and hepatic histopathological alterations; (2) compositional shifts in gut microbiota and associated metabolic derivatives. This work offers insights into the supplementation of FO and BO in diet in the amelioration of obesity and its related symptoms.

## 2. Materials and Methods

### 2.1. Preparation of Blended Oil and Animal Diets

Flaxseed oil was supplied by Shengmai Industrial Co., Ltd. (Liaoning, China). Sunflower and olive oils were obtained from COFCO Fortune Food Sales & Distribution Co., Ltd. (Tianjin, China). The production processes of flaxseed oil, sunflower oil, and olive oil all adopt the cold-pressing method. Specifically, the olive oil used is extra virgin olive oil. As outlined by the World Health Organization (WHO), saturated fatty acids (SFAs) should be replaced with PUFAs in the diet and the total intake of SFAs should not exceed 10% E (percentage of energy supply). It also proposes an adequate linoleic acid intake of 2–3% E and recommends that total ω-3 fatty acid intake per day range between 0.5 and 2% E [26]. However, no single vegetable oil or fat in nature can meet these WHO guidelines. To address this, a blended oil composed of flaxseed, sunflower, and olive oil was designed by Matlab in the context of WHO guidelines in this study. Their mass ratio (flaxseed/sunflower/olive oil) was set as 11.90:51.64:36.46 (*w*/*w*/*w*). The blended oil’s fatty acid composition was 9.1% palmitic acid (C16:0), 3.7% stearic acid (C18:0), 44.83% oleic acid (C18:1), 35.05% linoleic acid (C18:2), and 7.32% α-linolenic acid (C18:3). In this study, the ratio of ω-6/ω-3 in FO was 0.75, the ratio of ω-6/ω-3 in BO was 0.78, and the ratio of ω-6/ω-3 was 4.79. Recent studies tend to favor a lower ω-6/ω-3 ratio (1:1) to reduce the risks of inflammation, obesity, and chronic diseases. However, the WHO still maintains a safe range of 4–5:1, as a complete 1:1 ratio is difficult to achieve through the daily diet. The ω-6/ω-3 ratio of BO in this study met the requirements of the WHO. Oils were combined in a beaker and subjected to magnetic agitation at 1000 r/min while maintaining the temperature at 30 °C for a duration of 20 min.

The animals were fed with a normal diet (ND), high-fat diet (HFD), flaxseed oil intervention diet (HFD+FO, half of the lard in HFD was replaced with flaxseed oil), and blended oil intervention diet (HFD+BO, half of the lard in HFD was replaced with blended oi) [27]. These experimental diets were customized by the Biopike Company (Chengdu, China). Various diet compositions and energy densities are recorded in Table 1.

### 2.2. Animals and Modeling

Five-week-old male C57/B6 mice (SPF) were obtained directly from Gempharmatech Co., Ltd. (Jiangsu, China). Forty-eight mice were housed in groups of four and maintained under regulated cage conditions (temperature: 23 ± 2 °C; humidity: 50 ± 10%; light cycle: 12 h light/dark)The animals were random distribution among four experimental groups, normal diet (ND, *n* = 12), high-fat diet (HFD, *n* = 12), flaxseed oil intervention diet (HFD+FO, *n* = 12), and blended oil intervention diet (HFD+BO, *n* = 12), and were maintained on their respective diets for 13 weeks. The food intake and body weight of each murine subject was quantitatively assessed on a weekly basis throughout the study period. Following the completion of the experiment, mice were cervically dislocated under sodium pentobarbital anesthesia. Blood was drawn from the retro-orbital nerve clusters, and the serum was obtained by centrifugation (3000× *g*, 10 min, 4 °C). Meanwhile, the tissue samples were stored at −80 °C. All animal experiments were performed in strict compliance with protocols approved by the Institutional Animal Care and Use Committee of Nanchang University (license No: NCULAE-20221030038).

### 2.3. Measurement of Fasting Glycemia and Serum/Liver Biochemical Indexes

Fasting glycemia was quantified at week 12. After a 12 h fast, blood was collected via the tail vein and glucose concentration was determined.

Serum lipid profiles (total cholesterol (TC), triglyceride (TG), high-density lipoprotein cholesterol (HDL-C), low-density lipoprotein cholesterol (LDL-C), aspartate aminotransferase (AST), and alanine aminotransferase (ALT)) were quantified using commercial assay kits, which were purchased from Nanjing Jiancheng Bioengineering Institute, (Nanjing, China). The levels of serum leptin (LEP) were detected by an ELISA kit purchased from Boster Biological Technology Co., Ltd. (Wuhan, China).

Liver tissue was homogenized with saline, and then the supernatant fraction was carefully aspirated and collected (12,000× *g* at 4 °C for 10 min) for further biochemical index measurement. Superoxidase dismutase (SOD) and malondialdehyde (MDA) were measured with commercial kits from Beyotime Biotechnology Co., Ltd. (Shanghai, China).

### 2.4. Histopathological Analysis

The liver and epididymal adipose tissues, after fixation, were processed through paraffin embedding, sectioning, and ethanol-based dehydration. Hematoxylin and eosin (H&E) staining was then applied to the prepared sections. Histopathological evaluations were carried out employing an inverted microscope from Nikon Corporation (Tokyo, Japan). The histology of the liver can be scored through the NAFLD activity score (NAS), as shown in Table 2 [28].

### 2.5. Assessment of SCFAs in Colon Contents

Mix the contents of the colon with normal saline, perform homogenization treatment (at 70 Hz for 180 s), then conduct centrifugation (at 2000 revolutions per minute for 10 min). Pass the supernatant through a hydrophilic filter membrane, add sulfuric acid solution, internal standard solution (dihexylbutyric acid), and ether, perform vortex mixing, let it stand for 20 min before centrifugation again, then pass the supernatant through a hydrophobic membrane filter, and wait for analysis. The GC analysis was performed by an Agilent 8890 GC chromatograph furnished with an HP-FFAP capillary column. Sample loading was conducted with a precise injection volume of 2 μL. The GC temperature protocol was optimized as follows: the initial isothermal phase at 80 °C for 0 min, followed by a 10 °C/min ramp to 190 °C, concluding with a 0.5 min isothermal period, followed by a rapid increase to 230 °C at 40 °C/min with a 4 min hold. Equilibration and post-run times were 1 min and 3 min, respectively. A split injection technique was utilized with a 15:1 split ratio for sample introduction. Nitrogen served as the carrier gas. The inlet and detector temperatures were maintained at 220 °C. Additional gas flow rates were optimized as follows: air at 400 mL/min, hydrogen at 30 mL/min, and makeup gas (nitrogen) at 30 mL/min.

### 2.6. Gut Microbiota

Fecal DNA was prepared through the MagBeads FastDNA Kit for Soil purchased from MP Biomedicals, LLC (Irvine, CA, USA), and the V3 and V4 hypervariable regions of prokaryotic 16S rDNA were amplified by PCR. The PCR amplification products were detected by 2% agarose gel electrophoresis and quantified by fluorescence using the Quant-iT PicoGreen dsDNA kit (Thermo Fisher Scientific, Waltham, MA, USA). Based on these findings, the samples were proportionally combined and subjected to sequencing on the Illumina MiSeq platform ( Illumina, San Diego, CA, USA). OTU clustering was conducted with USEARCH (v7.0), followed by α- and β-diversity analyses in QIIME2 (v1.9.1). β-Diversity patterns were visualized via PCoA using a Bray–Curtis distance-based approach.

### 2.7. Data Analysis

Data are presented as mean ± SD. Statistical analyses were conducted using GraphPad Prism 10 and IBM SPSS Statistics 26. Statistical significance was determined using one-way ANOVA followed by Duncan’s multiple range test, and *p* < 0.05 was considered significant.

## 3. Results

### 3.1. Flaxseed Oil and Blended Oil Alleviated Body Weight Gain and Fat Accumulation in HFD-Fed Mice

**Figure 1:**
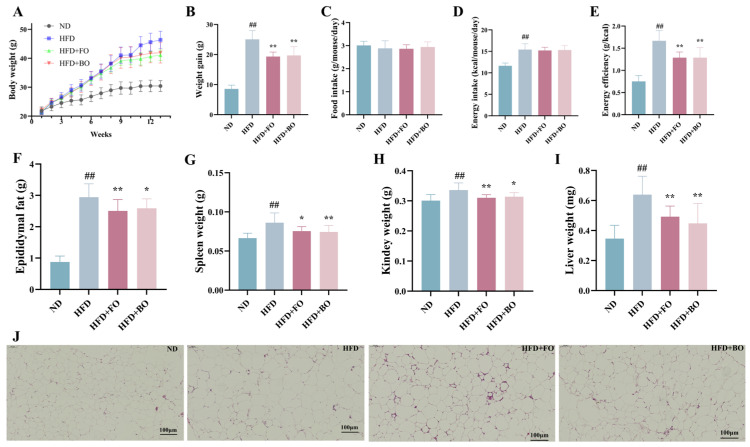
Effects of FO and BO on body weight gain and fat accumulation in HFD-induced obese mice. (**A**) Body weight. (**B**) Body weight gain. (**C**) Food intake. (**D**) Energy intake. (**E**) Energy efficiency. (**F**) Epididymal fat weight. (**G**) Spleen weight. (**H**) Kidney weight. (**I**) Liver weight. (**J**) H&E staining of epididymal fat (scale bar, 100 μm). (*n* = 12). ^##^ *p* < 0.01 compared with the ND group; * *p* < 0.05 and ** *p* < 0.01 compared with the HFD group. The following symbols are the same.

Mice in the HFD group developed dietary obesity and gained more significant weight compared to the ND group (Figure 1A,B). When half of the lard in HFD was replaced by flaxseed or blended oil (HFD+FO/HFD+BO group), results demonstratedthat flaxseed or blended oil had significant anti-obesity effects in HFD mice (multiple comparisons of weight gain on week 13: 8.57 ± 1.25 g in the ND group; 25.08 ± 2.96 g in the HFD group; 19.35 ± 1.47 g/19.71 ± 2.96 g in the HFD+FO/HFD+BO group). Meanwhile, both the HFD+FO and HFD+BO groups exhibited significantly reduced epididymal fat, spleen, kidney, and liver weights compared to the HFD group (Figure 1F,I) (*p* < 0.05). No notable disparity in food intake was observed between group means during a 13-week feeding period, but mice in the HFD, HFD+FO, and HFD+BO groups obviously took in more energy due to higher fat intake (Figure 1C,D). FO and BO intervention markedly decreased the energy efficiency in HFD-fed mice (Figure 1E), suggesting that the weight reduction effect of FO and BO may be related to the decreased energy efficiency of FO and BO diets. Epididymal adipose tissue in mice was stained with hematoxylin–eosin to observe the extent of pathology. The HFD group exhibited a larger volume of epididymal adipose tissue, and this enlargement of adipocytes was mitigated by flaxseed or blended oil (Figure 1J). These results suggest that flaxseed or blended oil could alleviate the HFD-induced obesity in mice.

### 3.2. Flaxseed Oil and Blended Oil Lowered Serum Lipid and Blood Glucose Levels in HFD-Fed Mice

**Figure 2:**
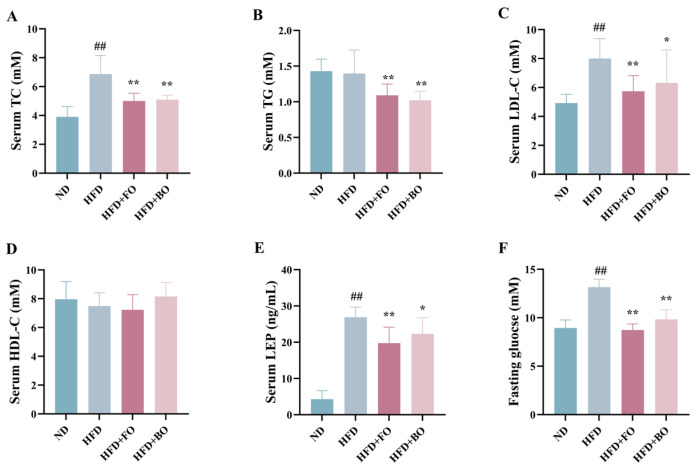
Effects of FO and BO on serum biochemical parameters in HFD-induced obese mice. (**A**) TC. (**B**) TG. (**C**) LDL-C. (**D**) HDL-C. (**E**) LEP. (**F**) Fasting glucose (*n* = 12). ^##^ *p* < 0.01 compared with the ND group; * *p* < 0.05 and ** *p* < 0.01 compared with the HFD group.

Numerous investigations have established that obesity is always accompanied by dyslipidemia [29,30]. Figure 2 demonstrates that HFD consumption induced significant dyslipidemia in the experimental mice, TC and LDL-C concentrations demonstrated statistically significant elevations (*p* < 0.01) (Figure 2A–D). FO and BO exhibited varying degrees of efficacy in restoring lipid profiles in HFD mice. TC, TG, and LDL-C levels were evidently decreased after the intake of FO (*p* < 0.01), and corresponding effects were noted in the HFD+BO group. Both FO and BO interventions markedly decreased serum leptin levels (Figure 2E). Moreover, fasting glucose concentrations were quantified in mice, it was found that the HFD triggered higher blood glucose levels (*p* < 0.01), and both FO and BO intervention effectively mitigated the HFD-induced hyperglycemia (*p* < 0.01), suggesting that FO and BO could regulate glucose homeostasis by suppressing the increase in fasting blood glucose levels (Figure 2F).

### 3.3. Flaxseed Oil and Blended Oil Ameliorated Hepatic Steatosis and Oxidative Stress in HFD-Fed Mice

**Figure 3:**
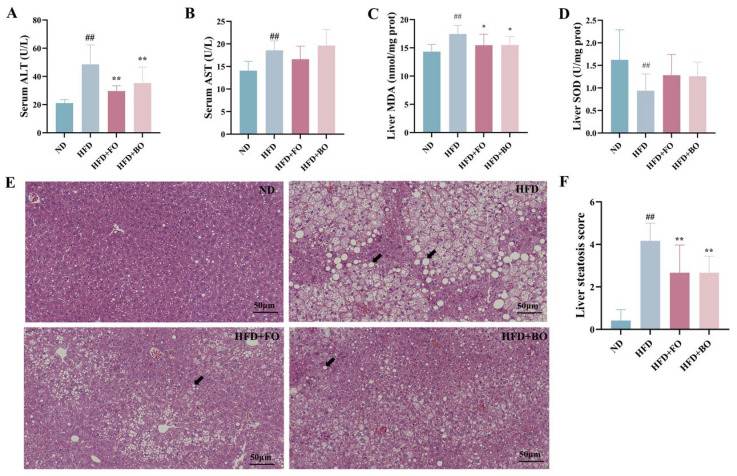
Effects of FO and BO on hepatic steatosis and oxidative stress in HFD-induced obese mice. (**A**) ALT. (**B**) AST. (**C**) Hepatic MDA content. (**D**) Hepatic SOD content. (**E**) H&E staining of liver (scale bar, 50 μm). (**F**) Liver steatosis score (*n* = 12). The black arrow indicated the rounded vesicle. ^##^ *p* < 0.01 compared with the ND group; * *p* < 0.05 and ** *p* < 0.01 compared with the HFD group.

Liver damage is potentially associated with excessive oxidative stress, as evidenced by significantly elevated serum ALT and AST levels, along with increased liver MDA concentrations in HFD mice. (*p* < 0.01) and FO and BO intervention significantly decreased serum ALT (*p* < 0.01) and liver MDA (*p* < 0.05) levels. The hepatic SOD levels in the HFD group demonstrated significant reductions (*p* < 0.01). Both FO and BO reversed this trend in hepatic SOD to varying extents (Figure 3A–D).

Figure 3E,F show the H&E-stained sections of mice liver. The hepatocytes of HFD-fed mice presented blurred outlines, irregular cell morphology, many rounded vesicles of varying sizes in the cytoplasm, hepatic steatosis (indicated by the black arrows), the displacement of nuclei to the cell edges, and disorganization of the hepatic lobules. The HFD+FO and HFD+BO groups showed obvious improvement in hepatic lesions, characterized by a reduced number of vacuoles, an intact cytoplasm, and clear nuclei.

### 3.4. Flaxseed Oil and Blended Oil Promoted the Production of SCFAs in the Colon Contents

**Figure 4:**
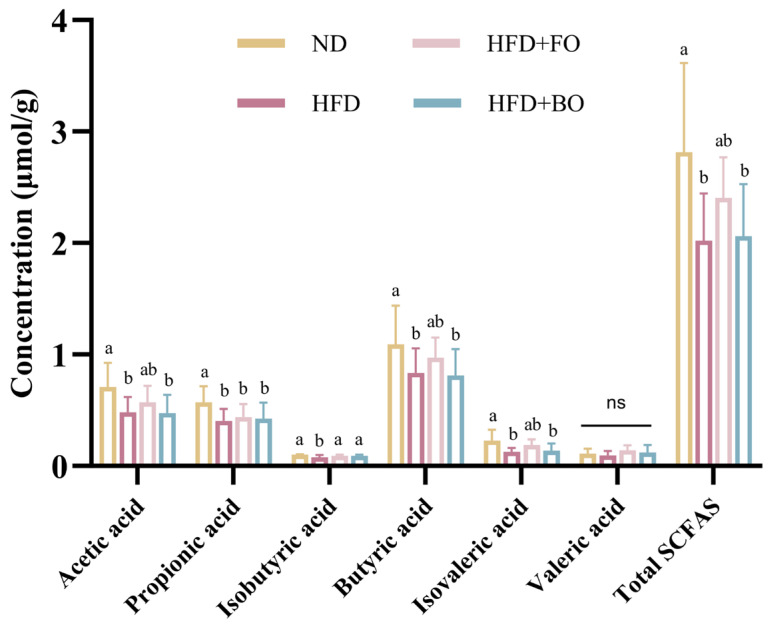
Effects of FO and BO on fecal SCFA concentrations in HFD-induced obese mice. Different letters showed statistically significant differences (*p* < 0.05). ns is not significant different.

SCFAs serve as key metabolites in host metabolism [31]. SCFAs exerted regulatory effects on both host lipid metabolism and gut microbiota [32]. The major SCFAs detected in colon contents were acetic, propionic, and butyric acid, and minor contents of isobutyric, valeric, and isovaleric acids were detected. The concentrations of individual SCFAs and the HFD-exposed cohort exhibited statistically significant reductions in total SCFA levels. In contrast, FO intake restored acetic, isobutyric, butyric, and isovaleric acid and total SCFA levels, while BO had a minimal impact on the SCFA levels (Figure 4).

### 3.5. Flaxseed Oil and Blended Oil Ameliorated the HFD-Induced Gut Microbiota Dysbiosis

**Figure 5:**
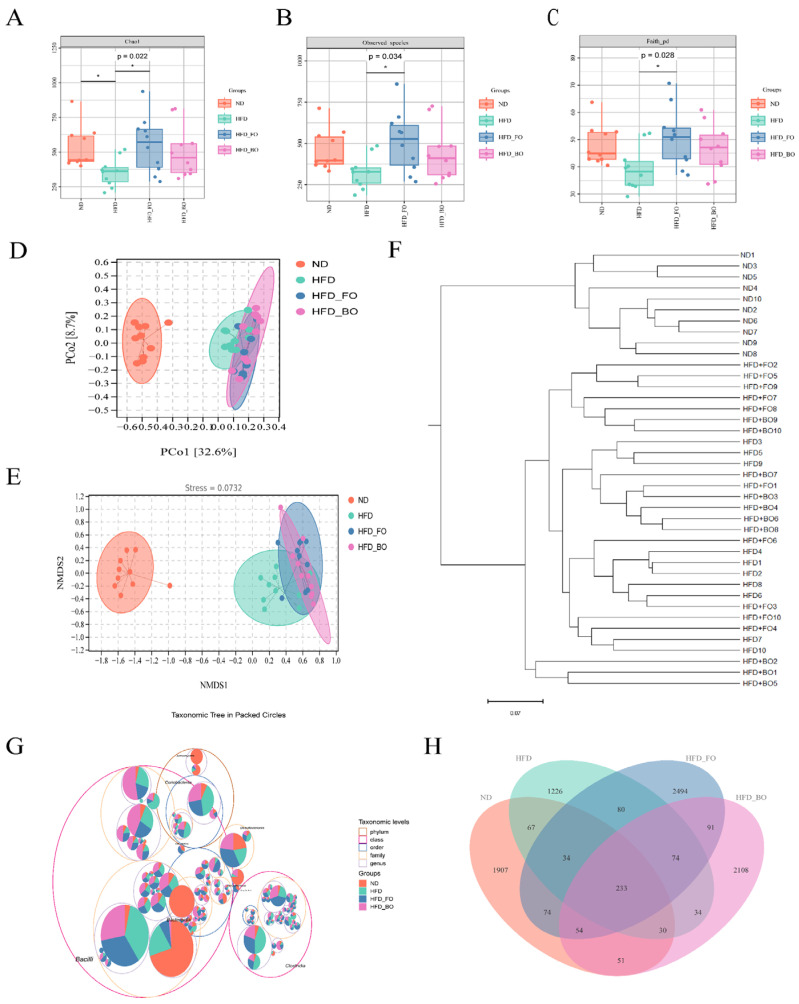
Effects of FO and BO on gut microbiota diversities in HFD-fed mice. Alpha diversity index, including Chao1 (**A**), Observed_species (**B**), Faith_pd indexes (**C**), and PCoA analysis (confidence level 90%) (**D**). NMDS analysis (**E**), hierarchical cluster analysis (**F**), classification level tree (**G**), and Venn diagram (*n* = 10) (**H**) are presented. * *p* < 0.05 compared with the HFD group.

**Figure A1:**
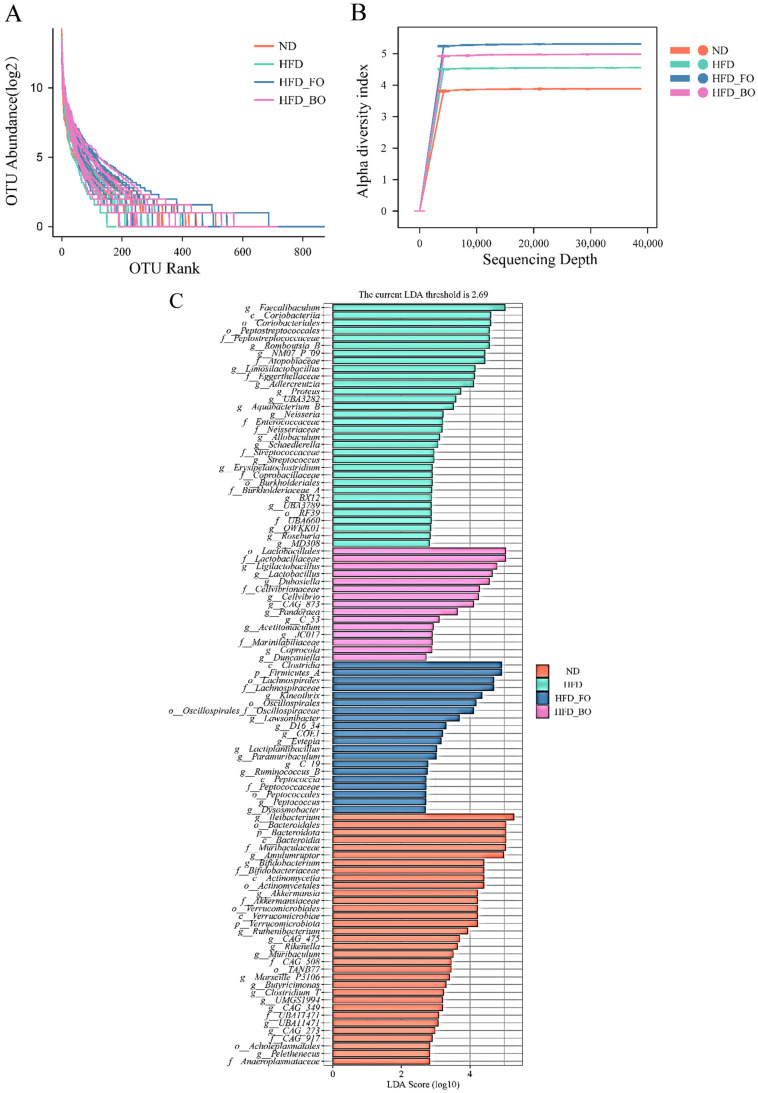
Effects of FO and BO on gut microbiota in HFD-fed mice. (**A**) Rank–abundance curve. (**B**) Species rarefaction curve. (**C**) LDA (*n* = 10).

The findings reveal that FO and BO accelerated the synthesis of SCFAs in colon contents, and the regulatory role of FO and BO in the gut microbiota was further investigated. Current studies have verified a significant correlation between the composition of gut microbiota and obesity. The intervention of gut microbiota through diet can reconstitute the balance of the intestinal microecology, enhance the intestinal barrier function, and strengthen its protective effect, thereby effectively preventing obesity and synergistically treating obesity-related metabolic disorders [33,34]. The abundance rank curves, which reflect the richness and evenness of community composition across samples, show wide and flat curves, indicating a rich and even species composition (Figure A1A). The degree of flattening of the sparse curve reflects the extent of the sequencing depth’s influence on sample diversity, and Figure A1B shows that the curve flattens as the sample size increases, demonstrating that the sequencing data accurately represents the diversity within the samples. Chao 1, observed species, and Faith’s PD evaluated the impact of FO and BO on gut microbiota alpha diversity (Figure 5A–C). After HFD feeding, the richness (Chao1 and observed species) and phylogenetic diversity (Faith’s PD) levels were lower than those in a normal diet, demonstrating that HFD reduced the α-diversity of the intestinal microbial community. The richness and phylogenetic diversity of gut microbes increased in the HFD+FO and HFD+BO groups. PCoA, NMDS, and hierarchical cluster analysis showed β-diversity differences among microbial communities in different groups. A Bray–Curtis distance analysis of PCoA revealed that the clustering of the gut microbiome was significantly different for each treatment group (Figure 5D). After HFD feeding, the HFD group was easily distinguished from the ND group. NMDS indicated that the mouse model was reliable, and the results were usable (Figure 5E). Hierarchical cluster analysis demonstrated substantial disparities among the experimental groups, particularly between the HFD group and the ND group, which were distinctly segregated. Furthermore, the dietary consumption of FO and BO exhibited pronounced differences in comparison with the high-fat diet intervention, and the extent of such differentiation exhibited varying degrees (Figure 5F). The circular hierarchical tree diagram depicts microbial community structure, with the outermost ring showing phylum-level taxa and inner rings representing genus- and species-level classifications The top 100 operational taxonomic units (OTUs) ranked by abundance are indicated by the innermost points. The area of each circle reflects the relative abundance of the corresponding taxonomic unit, while the area of the sectors in the pie chart is positively correlated with the abundance of the corresponding taxonomic unit within each group. Figure 5G reveals that the largest circle represents the Bacilli class, which belongs to the Firmicutes phylum within Gram-positive bacteria. It indicates that this microbial community holds a dominant position in the intestinal tract, and its abundance in the sample is significantly higher than that of other taxonomic units, resulting in a larger circle at the class level compared to the phylum level. The specific species that contribute to alterations in microbial community composition were subjected to further investigation. As illustrated in Figure 5H, the region of color block overlap represents the common ASV/OTU (amplicon sequence variant/operational taxonomic unit) among groups, with a total of 233 ASV/OTUs observed across all groups. The unique ASV/OTUs for the ND, HFD, HFD+FO, and HFD+BO groups were 1907, 1226, 2494, and 2108, respectively. In contrast to ND, the HFD led to a significant decline in the number of operational taxonomic units (OTUs). The findings indicate that FO and BO mitigated the decline in OTU numbers resulting from HFD and augmented the diversity of the intestinal microbial community in HFD mice.

**Figure 6:**
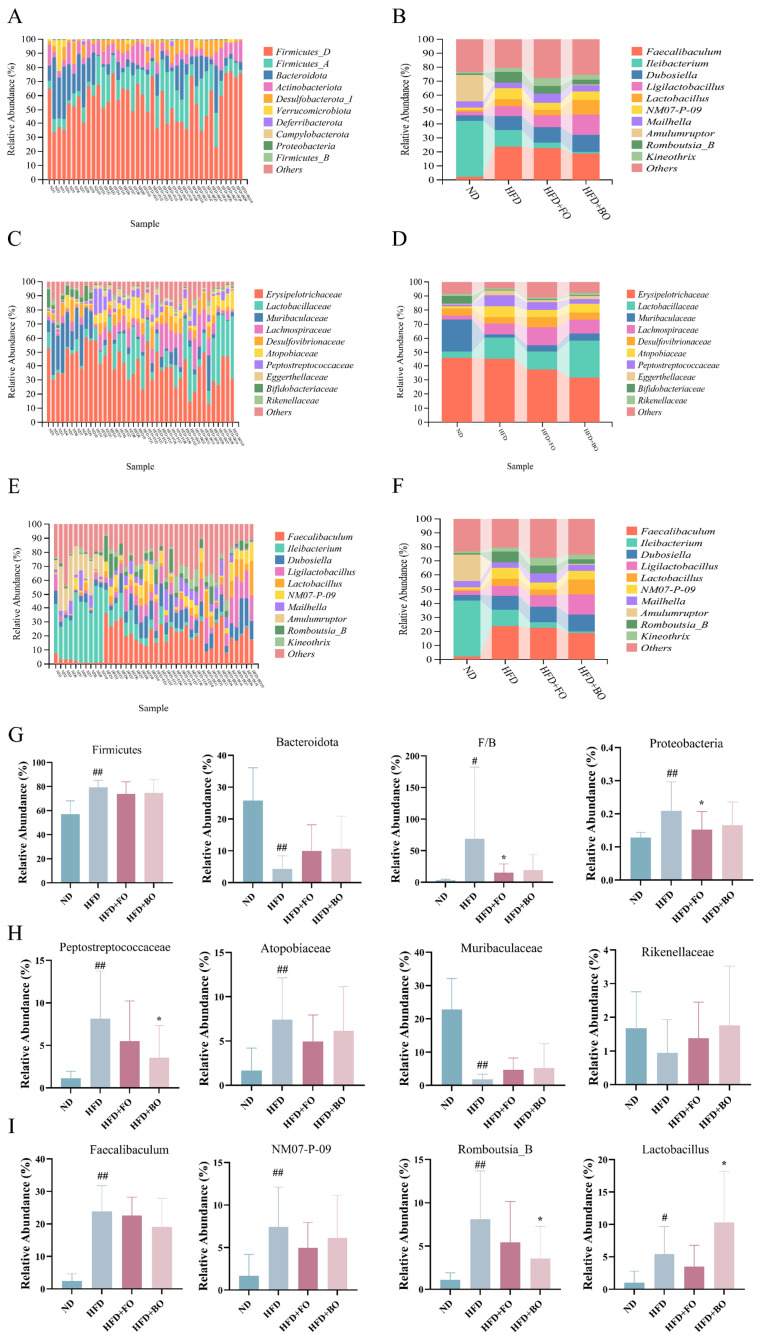
Effects of FO and BO on the composition of the gut microbiota at different classification levels. (**A**,**B**,**G**) Phylum level. (**C**,**D**,**H**) Family level. (**E**,**F**,**I**) Genus level (*n* = 10). ^#^ *p* < 0.05 and ^##^ *p* < 0.01 compared with the ND group; * *p* < 0.05 compared with the HFD group.

Figure 6A,B demonstrate the phylum level. The gut microbiota mainly consisted of Firmicutes_D, Firmicutes_A, and Bacteroidota, with Firmicutes_D being the dominant species. FO and BO alleviated the increase in Firmicutes and Proteobacteria and the decrease in Bacteroidota caused by HFD feeding. Taxonomic analysis showed that the Firmicutes/Bacteroidota ratio (F/B ratio) increased substantially after HFD feeding, while both FO and BO decreased the F/B ratio (Figure 6G). At the family level, the abundance of Atopobiaceae and Peptostreptococcaceae in the HFD group was elevated, while the levels of Muribaculaceae and Rikenellaceae were decreased. However, these changes were reversed after the intake of FO and BO (Figure 6C,D,H). At the genus level, *Faecalibaculum*, *Ileibacterium*, *Lactobacillus*, and NM07-P-09 were the dominant genera of bacteria. In contrast to the ND group, the contents of *Faecalibaculum*, NM07-P-09, and *Romboutsia_B* in the HFD group were markedly elevated, but FO and BO supplementation offset this change to a certain extent. Meanwhile, the HFD+BO group exhibited greater *Lactobacillus* abundance (Figure 6E,F,I).

**Figure 7:**
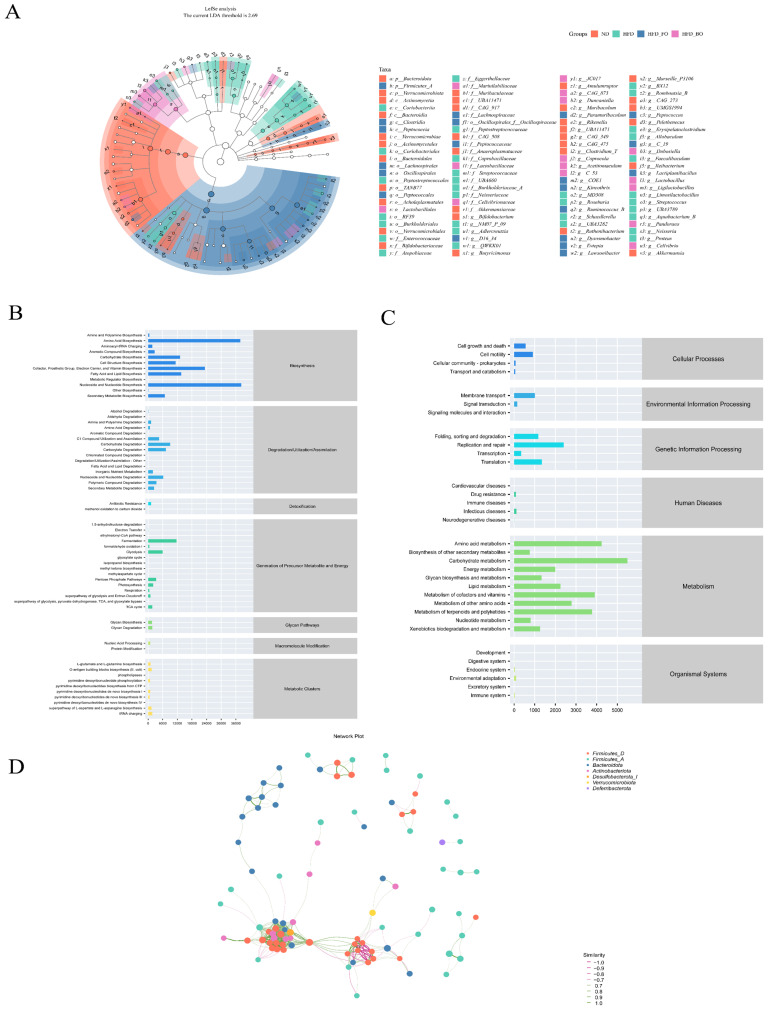
Effects of FO and BO on species differentiation and species specificity in HFD-fed mice. (**A**) LefSe analysis. (**B**) MetaCyc analysis. (**C**) KEGG analysis. (**D**) Association network (*n* = 10).

LEfSe analysis identified differences in the compositional structure of the gut microbiota between groups and looked for biomarkers that were statistically different. The taxonomic cladogram (Figure 7A) and the LDA histogram (Figure A1C) clearly showed that the dominant strains of the ND group were p_Bacteroidota, p_Verrucomicrobiota, and c_Actinomycetia. The HFD group was mainly composed of f_Peptostreptococcaceae, c_Coriobacteriia, and o_Coriobacteriales. The HFD+FO group was dominated by c_Clostridia, *g_Lactiplantibacillus*, and *g_Ruminococcus_B*, while the HFD+BO group was dominated by *g_Lactobacillus*, *g_Dubosiella*, and *g_Coprocola*. Meanwhile, the correlation network diagram of microbial ecology shows that Firmicutes and Bacteroidetes were the most abundant in this study (Figure 7D), which is in line with the above experimental results.

### 3.6. Prediction of Gut Microbial Metabolism Using PICRUSt2

The aforementioned analyses emphasize the diversity and species composition of the gut microbiota. To conduct a more in-depth study on the metabolic capabilities of the microbiota, potential metabolic pathways were predicted by analyzing the gene sequences of all samples using the MetaCyc and KEGG databases. The MetaCyc database showed the highest relative abundance of metabolic pathways associated with biosynthesis, degradation/utilization/assimilation, and the generation of precursor metabolites and energy (Figure 7B). The categorization of function prediction was mainly associated with metabolic pathways, including carbohydrate, amino acid, energy (lipid metabolism and cofactors and vitamins metabolism), genetic information processing, and cellular processes (Figure 7C).

## 4. Discussion

Obesity now represents a worldwide health crisis [35], and the associated weight gain contributes to a variety of chronic diseases, encompassing cardiovascular disease, type 2 diabetes, NAFLD, and metabolic disorders [36]. Current therapeutic drugs for obesity, such as orlistat capsules and metformin hydrochloride tablets, demonstrate limited efficacy and can produce serious adverse effects [37]. Therefore, developing safer and more efficient solutions to improve obesity is extremely pressing. In the recent period, dietary therapy is considered an effective alternative for reducing obesity and associated metabolic complications [38]. This study focused on exploring the role of the ω-3 PUFA-rich diet, achieved by FO and BO supplementation, on HFD-induced obesity. The data revealed that both FO and BO relieved obesity symptoms, ameliorated weight gain and lipid accumulation, lowered blood glucose levels, mitigated liver damage, resisted oxidative stress, and regulated gut microbiota and their metabolites.

Although there are complex causes for the development of obesity, the chronic consumption of high-energy foods is a primary contributor to the condition [39]. Studies consistently indicate that obesity results from an imbalance in energy metabolic processes. In our study, it could be seen that the inclusion of FO and BO in the feed was able to significantly reduce the increase in body weight and tissue organ weights in HFD-fed mice, and FO had a more significant effect on improving the weight of epididymal fat and kidneys, while BO had a more significant effect on improving the weight of the spleen. The data harmonized with previous study outcomes [21]. Our results indicate that FO and BO can significantly reduce energy efficiency, and the reason might be the ALA abundant in FO and BO. It has been shown that the consumption of foods rich in ω-3 PUFAs decreases energy efficiency compared to lard [40]. Therefore, decreasing energy efficiency may be one of the potential mechanisms by which FO and BO alleviate obesity.

Obesity is frequently linked to excessive lipid accumulation and abnormal glucose metabolism [41]. When caloric intake consistently exceeds expenditure, excess energy is converted into triglycerides and stored in fat cells, causing their hypertrophy. [42]. As a result, ectopic fat deposits might occur in the internal organs and muscles, leading to metabolic disorders and other health problems [43]. In this study, we observed fat deposition in the epididymis and liver. HFD resulted in fat synthesis and an increased volume of adipocytes, promoting an increase in white fat weight, such as epididymal fat. Studies have shown that ω-3 PUFAs can reduce fat production, accelerate fat breakdown, and reduce lipid storage and adipocyte size [44].

This study indicates that FO and BO intervention can effectively suppress elevated serum TC, TG, and LDL-C levels in HFD mice. Moreover, FO showed a better improvement effect on LDL-C than BO, thereby effectively lowering the risk of hyperlipidemia and atherosclerosis. Simultaneously, the research also found that after supplementation with FO and BO, the level of leptin in the serum decreased. According to the positive correlation between adipocyte volume and blood leptin concentration established by Skurk et al. [45], this study suggests that FO and BO may suppress adipocyte hypertrophy by reducing leptin secretion. The dysregulation of lipid metabolism constituted a central pathological feature in HFD-induced metabolic dysfunction. Our observations of characteristic hyperlipidemia in HFD mice—marked by elevated TC, TG, and LDL-C levels—align with Tian et al.’s [46] findings on adipokine dysregulation secondary to aberrant adipocyte proliferation. The excessive release of pathogenic adipokines and pro-inflammatory cytokines disrupts lipolytic–lipogenic homeostasis [47], while elevated LDL-C contributes to vascular plaque formation through oxidative modification, a critical driver of atherogenesis [48]. At the molecular level, FO and BO modulated lipid metabolism through multiple pathways: the PPARα-mediated enhancement of fatty acid oxidation as demonstrated in flaxseed oil studies [49], coupled with FAS expression inhibition to limit lipid accretion [50]. These coordinated mechanisms collectively preserve lipid homeostasis, establishing a scientific rationale for FO and BO supplementation in metabolic disorder management. Since impaired glucose metabolism is closely linked to the development of type 2 diabetes [51], we also explored the implications of FO and BO on blood glucose in obese mice. The results showed that both the HFD+FO and HFD+BO groups exhibited significantly lower fasting blood glucose levels compared to the HFD group, suggesting that FO and BO can reduce the likelihood of obesity-induced type 2 diabetes.

In this study, HFD-fed mice exhibited hepatic steatosis, lipid deposition, and severe hepatic tissue damage, while dietary supplementation with FO and BO significantly attenuated liver injury, ameliorated oxidative stress, and alleviated hepatic steatosis, and the improvement effect of BO was stronger than that of FO. The liver maintains normal physiological functions by regulating lipid metabolism and glucose cycle homeostasis, and mitigating oxidative stress [52,53]. NAFLD is a widespread condition associated with obesity, characterized by steatosis and accompanied by reactive oxygen species overproduction and endoplasmic reticulum stress [54]. Chronic HFD consumption directly induces liver injury through oxidative stress [55], manifested by elevated serum ALT and AST activities [56], concomitant with decreased SOD activity and increased MDA levels [57]. The ameliorative effects of FO and BO on the aforementioned biomarkers suggest their potential to protect hepatic function through the modulation of redox homeostasis.

The gut microbiome’s influence on metabolic regulation and its association with disease development have gained significant attention [58,59]. It serves an important function in regulating the body’s energy metabolism and immune responses, with strong ties to obesity [60]. A notable reduction in the abundance and diversity of gut microbiota serves as a critical marker of gut microbiota dysbiosis [61,62,63]. Our study found that HFD induced a pronounced reduction in gut microbiota richness and diversity and a change in species composition in mice compared with a normal diet. When mice were supplemented with flaxseed oil rich in ALA, the community richness and diversity increased, and the species composition was improved. At the phylum level, an elevated F/B ratio leads to dysbiosis of the gut microbiota and is a well-recognized marker of HFD-induced obesity [64]. This study found that HFD intervention significantly increased the F/B ratio in the intestinal microbiota of the HFD group. The supplementation of FO and BO alleviated the imbalance of the microbiota structure. The results showed that FO and BO could reduce the increase in Firmicutes and Proteobacteria induced by HFD and increase the level of Bacteroidetes. FO was more effective than BO in reducing the level of Proteobacteria. An increase in the abundance of Firmicutes enhances the ion transport activity of intestinal epithelial cells, thereby improving energy acquisition efficiency and promoting adipocyte hypertrophy. In contrast, Bacteroidetes are essential for preserving colonic mucosal barrier integrity by activating the propionate-mediated intestinal gluconeogenesis pathway, and this mechanism was biologically associated with the improved glucose regulation ability in the HFD+FO and HFD+BO groups. In addition, the distribution of Gram-negative pathogens within *Proteobacteria* was positively correlated with LPS synthesis [64]. The study by Kim et al. [65] demonstrated that HFD enhanced both Proteobacteria numbers and LPS content within the intestinal microbiota of mice. Mechanistic studies have established that LPS, a key virulence factor of Gram-negative bacteria, initiates the TLR4/MAPK signaling cascade, driving the transcriptional activation of pro-inflammatory cytokine genes through the phosphorylation of JNK and p38 kinases. This signaling axis provokes sustained systemic inflammation, ultimately contributing to tissue damage and metabolic dysregulation [66]. At the family level, our experimental results demonstrate that Atopobiaceae and Peptostreptococcaceae (families positively correlated with obesity) were elevated in HFD-fed mice, while Muribaculaceae and Rikenellaceae (families associated with microbial balance) showed reduced abundance. Dietary supplementation of FO and BO is capable of ameliorating this condition. These findings align with previous studies: Atopobiaceae and Peptostreptococcaceae have been mechanistically linked to pro-inflammatory responses and metabolic dysregulation [67,68], whereas Muribaculaceae and Rikenellaceae are recognized as key contributors to gut microbial diversity and metabolic homeostasis through their roles in bile acid metabolism and mucosal integrity maintenance [69,70]. At the genus level, FO and BO could lower the level of *Faecalibaculum*, with BO was stronger than FO, and BO could significantly increase the *Lactobacillus* content. At the same time, FO and BO also inhibited the HFD-induced increase in *Romboutsia_B* and NM07-P-09 of *Actinobacteriota*. *Faecalibaculum* is observed to be related to elevated lipid levels (TG, LDL-C, and HDL-C) and has the potential to contribute to cardiovascular-related diseases [71]. He et al. [72] noted that in the genus *Faecalibaculum*, *F. rodentium* was enriched in HFD-fed mice. *Lactobacillus* can promote the formation of SCFAs, and some lactic acid bacteria also have a hypoglycemic effect [73,74]. Our LEfSe analysis revealed that Bacteroidota was identified as the characteristic phylum in the ND group, with its abundance considerably diminished in obese individuals. The HFD group showed a dominance of Peptostreptococcaceae, while FO supplementation notably increased the relative levels of Clostridia. Additionally, FO and BO differentially modulated *Lactobacillus* populations: FO enriched the *g_Lactiplantibacillus*, whereas BO enhanced *g_Lactobacillus* abundance. The inverse correlation between Bacteroidota depletion and obesity has been validated in prior studies [75,76]. The overrepresentation of Peptostreptococcaceae in HFD-fed mice may exacerbate metabolic dysregulation via gut barrier dysfunction. Specifically, Peptostreptococcaceae-driven intestinal hyperpermeability (“leaky gut”) facilitates the systemic translocation of pro-inflammatory mediators like LPS, triggering chronic low-grade inflammation and accelerating obesity progression [77,78]. Notably, FO-induced Clostridia enrichment aligns with butyrate biosynthesis activation, a key SCFA that suppresses adiposity by enhancing intestinal epithelial energy metabolism [79]. Furthermore, *Lactobacillus* proliferation not only reduces blood glucose via lactate production [73,74] but also maintains gut microecological homeostasis through the competitive exclusion of pathogenic bacteria [80]. The findings highlighted that HFD caused the disruption of gut microbiota balance, and harmful bacteria dominated, and FO and BO restored HFD-induced gut microbiota imbalance in mice. However, the anti-obesity effects of FO and BO in the body involve a multi-factor regulatory network. In the future, the key role of the intestinal microbiota in this model can be verified through methods such as fecal microbiota transplantation, and the mechanisms of FO and BO in obesity can be further clarified by using gene knockout experiments, metagenomic sequencing, RNA sequencing, and other studies.

## 5. Conclusions

This study assessed the efficacy of FO and BO in alleviating HFD-induced obesity in mice. After 13 weeks of dietary supplementation with FO or BO, significant reductions in body weight, organ index, and epididymal fat accumulation were observed. Additionally, glucose intolerance and dyslipidemia were improved, and liver tissue was protected. FO and BO supplementation also restored gut microbiota diversity and the Firmicutes/Bacteroidetes (F/B) ratio, which were disrupted by HFD. An increased abundance was observed of key bacteria associated with obesity prevention, such as Bacteroidetes, Clostridia, and Lactobacillus, while the proliferation of obesity-related bacteria, including Firmicutes, Proteobacteria, and Peptostreptococcaceae, was suppressed. Furthermore, the production of SCFAs was regulated. These results present the possible role of FO and BO in mitigating obesity-related pathophysiological manifestations and metabolic dysregulation and may serve as a foundation for future studies on the practical use concerning flaxseed oil resources. Although this study demonstrated the above conclusions, it is necessary to acknowledge several limitations, including the 13-week study duration (which may have missed long-term effects), the lack of in vivo mechanism validation, and the study subjects being of a single gender. Future research should address these issues by extending the study period, conducting in vivo studies, and adopting a gender-balanced design.

## Figures and Tables

**Table 1:** Compositions of experimental diets.

Ingredients (g)	ND	HFD	HFD+FO	HFD+BO
Casein	200	200	200	200
L-Cystine	3	3	3	3
Corn Starch	506.2	0	0	0
Maltodextrin	125	125	125	125
Sucrose	68.8	68.8	68.8	68.8
Cellulose	50	50	50	50
Soybean Oil	25	25	25	25
Lard	20	245	122.5	122.5
Flaxseed Oil	0	0	122.5	0
Blended Oil	0	0	0	122.5
Mineral Mix	10	10	10	10
Dicalcium Phosphate	13	13	13	13
Calcium Carbonate	5.5	5.5	5.5	5.5
Potassium Citrate, 1 H_2_O	16.5	16.5	16.5	16.5
Vitamin Mix	10	10	10	10
Choline Bitartrate	2	2	2	2
Total	1055	773.8	773.8	773.8
Calories supplementation (kcal %)				
Proteins	20	20	20	20
Carbohydrates	70	20	20	20
Fats	10	60	60	60
Energy (kcal/g)	3.85	5.24	5.24	5.24

**Table 2:** The scoring index of liver tissue damage.

Steatosis	Lobular Inflammation	Hepatocellular Ballooning	Score
the presence of steatosis in <5% of hepatocytes	none	normal hepatocytes	0
5–33%	≤2 foci per 20×	the liver cells were arranged in clusters, the majority of the cells maintained a round shape, the cytoplasm was lightly stained and presented a reticular configuration, and there was no significant difference in cell size	1
34–66%	>2	symptoms were similar to score 1, but the liver cells were enlarged and their number increased by two times	2
>66%	\	\	3

## Data Availability

The original contributions presented in this study are included in the article. Further inquiries can be directed to the corresponding authors.
